# Ferroptosis-Related Proteins Are Potential Diagnostic Molecular Markers for Patients with Preeclampsia

**DOI:** 10.3390/biology11070950

**Published:** 2022-06-22

**Authors:** Meiting Shi, Xiaofeng Yang, Yuzhen Ding, Lu Sun, Ping Zhang, Mengyuan Liu, Xiaoxue Han, Zhengrui Huang, Ruiman Li

**Affiliations:** Department of Obstetrics and Gynecology, The First Affiliated Hospital of Jinan University, Guangzhou 510000, China; meitingshi2018@163.com (M.S.); yxf565548660@163.com (X.Y.); dingyuzhen8848@126.com (Y.D.); sunlu5562@163.com (L.S.); zhang44ping@gmail.com (P.Z.); mengyuan_s@163.com (M.L.); 13249144308@163.com (X.H.); hzhengr@126.com (Z.H.)

**Keywords:** preeclampsia, ferroptosis, bioinformatics, diagnosis

## Abstract

**Simple Summary:**

In this work, a number of key genes involved in ferroptosis were identified, which aimed to provide clues for further studying the role of ferroptosis in preeclampsia. Among these hub genes, we selected p53 and c-Jun for further validation based on the relationship between their expression levels and clinicopathologic features and diagnosis value using the ROC curve. Our study may provide interesting insight into the pathological mechanism of preeclampsia from the perspective of bioinformatics analysis.

**Abstract:**

Preeclampsia (PE) is the leading cause of maternal and fetal mortality and morbidity. Early and accurate diagnosis is critical to reduce mortality. Placental oxidative stress has been identified as a major pathway to the development of PE. Ferroptosis, a new form of regulated cell death, is associated with iron metabolism and oxidative stress, and has been suspected to play a role in the pathophysiology of PE, although the mechanism is yet to be elucidated. The identification of potential ferroptosis-related biomarkers is of great significance for the early diagnosis and treatment of PE. A gene expression dataset of peripheral blood samples was downloaded from the Gene Expression Omnibus (GEO) dataset. Differentially expressed genes (DEGs) were filtrated with the R package “limma”. Gene Ontology (GO) and Kyoto Encyclopedia of Genes and Genomes (KEGG) enrichment analyses of the DEGs were then conducted. Ferroptosis-related DEGs were screened by overlapping the ferroptosis-related genes with DEGs. The protein–protein interaction (PPI) network was used to identify the key ferroptosis-related DEGs. Enzyme-linked immunosorbent assay (ELISA) was used to validate changes in the selected key ferroptosis-related DEGs. The correlations between the key genes and clinical and pathological characteristics were analyzed. Finally, the diagnostic value of these key genes for PE was confirmed by a receiver operating characteristic (ROC) curve. A total of 5913 DEGs were identified and 45 ferroptosis-related DEGs were obtained. Besides, ferroptosis-related pathways were enriched by KEGG using DEGs. The PPI network showed that p53 and c-Jun were the critical hub genes. ELISA showed that p53 in the serum of PE patients was higher than that of the control group, while c-Jun was lower than that of the control group. Analysis of the clinicopathological features showed that p53 and c-Jun were correlated with the PE characteristics. Finally, based on the area under curve (AUC) values, c-Jun had the superior diagnostic power (AUC = 0.87, *p* < 0.001), followed by p53 (AUC = 0.75, *p* < 0.001). Our study identified that two key genes, p53 and c-Jun, might be potential diagnostic biomarkers of PE.

## 1. Introduction

Preeclampsia (PE), characterized by hypertension and proteinuria after 20 weeks of gestation, is a pregnancy-specific cardiovascular complication that affects 2 to 8% of all pregnancies and is the leading cause of maternal and fetal morbidity and mortality [1]. Although the etiology of PE has been extensively studied in the past few decades, its pathogenesis is still not fully understood [2]. Currently, the treatment of PE is limited to the control of hypertension and maternal–fetal monitoring, and timely termination of pregnancy remains the only effective method [3]. Therefore, early recognition, accurate diagnosis, and appropriate management of patients with PE are important to reduce the risk of adverse maternal and fetal outcomes. PE is now diagnosed based on hypertension and proteinuria, but this method lacks sensitivity and specificity, and it has a dismal prognosis for unfavorable maternal and fetal outcomes [4]. Hence, it is very necessary to identify new potential biomarkers for screening, diagnosis, and monitoring of PE.

The pathogenesis of PE is complex and involves many different mechanisms, including immune imbalance, vascular endothelial cell damage, and inflammatory processes [5]. The prevailing view is that abnormal invasion of trophoblasts may cause inadequate remodeling of the maternal spiral arteries, which leads to utero-placental high-resistance circulation [6], resulting in a state of ischemia and hypoxia in the placenta, which triggers an oxidative stress stage. Recently, a large number of studies showed that programmed cell death plays an important role in trophoblast injury and placental physiology [7,8]. Ferroptosis, an iron-dependent form of programmed cell death, has been identified in preeclampsia, but the mechanism of ferroptosis in the placenta remains unclear [7,9]. It is known that ferroptosis is morphologically, biochemically, and genetically distinct from other already established modes of cell death, including apoptosis, autophagy, and necrosis [10,11]. Moreover, oxidative stress and lipid peroxidation are two major causes for ferroptosis, characterized by the intracellular accumulation of reactive oxygen species, which is closely associated with the pathophysiology of PE [12]. It is a well-known fact that the placenta has been identified as the central organ in the pathogenesis of PE. Indeed, studies found that trophoblast cells may be more prone to ferroptosis than other cell types at the maternal–fetal interface due to the high iron, the high level of long-chain polyunsaturated fatty acids, and the high expression of Lpcat3 and Sat1 [13,14,15].

Multiple signaling pathways, including the mitogen-activated protein kinase (MAPK) signaling pathway, p53 signaling pathway, and the Hippo pathway, have been found to play important roles in ferroptosis [16,17,18,19]. In addition to the two classic ferroptosis-regulating genes, SLC7A11 and GPX4, a growing number of ferroptosis-related genes (FRGs) have been identified as contributors to some diseases [20,21,22,23,24]. As the research advanced, accumulating evidence indicates that ferroptosis is associated with a variety of human diseases, including carcinogenesis, degenerative diseases, cerebral hemorrhage, renal degeneration, and ischemia–reperfusion injury [25,26,27,28,29,30]. Recent studies have suggested that ferroptosis plays an important role in the development and progression of PE [31]. Therefore, identification of ferroptosis-related biomarkers for the early diagnosis of PE is vital and may contribute to early intervention in PE patients.

In this study, we used a data-mining approach to identify the differentially expressed genes (DEGs) in blood samples between pregnant women with preeclampsia and healthy pregnant women. Then, functional enrichment analyses showed these DEGs to be enriched in biological functions related to ferroptosis. Next, these DEGs were intersected with the ferroptosis dataset to acquire the ferroptosis DEGs. Finally, based on these screening results, many potential biomarkers were identified, and p53 and c-Jun were validated via enzyme-linked immunosorbent assay (ELISA) in the validation study. In conclusion, this study may provide potential biomarkers for the diagnosis of PE.

## 2. Materials and Methods

### 2.1. Acquisition and Processing of Gene Expression Data

The National Center for Biotechnology Information (NCBI) Gene Expression Omnibus (GEO) database (https://www.ncbi.nlm.nih.gov/geo/ (accessed on 2 May 2022)) is an international open-access data platform. The gene expression profiling dataset GSE149440, which is based on the GPL28460 Affymetrix Human Transcriptome Array 2.0 platforms, was downloaded from GEO. The dataset contains 735 samples composing of whole blood from the controls who delivered at term (*n* = 314) and patients with early-preeclampsia (*n* = 66), preterm premature rupture of membranes (PPROM) (*n* = 189), and spontaneous preterm delivery (sPTD) (*n* = 166). The data of the controls and patients with early-preeclampsia were further analyzed. Three types of ferroptosis-related genes were download from FerrDb (http://www.zhounan.org/ferrdb/legacy/index.html (accessed on 2 May 2022)), including 150 drivers, 109 suppressors, and 123 markers. In total, 214 genes were screened after removing non-human and duplicate genes.

### 2.2. Screening of the Differentially Expressed Genes (DEGs)

The differentially expressed genes between the controls and patients were screened using the “limma” R package. To obtain the significantly DEGs, a *p* value < 0.05 and log2 (Fold Change) ≠ 0 were set as the criteria of identification.

### 2.3. Analysis of Functional Enrichment and Pathway

In order to study the biologic functions and pathways of the DEGs, Gene Ontology (GO) and Kyoto Encyclopedia of Genes and Genomes (KEGG) enrichment analyses were performed. GO annotations of the genes in the R software package org.hs.eg.db (Version 3.1.0, Bioconductor) were used as the background to map genes into the background set, and the R package clusterProfiler (Version 3.14.3, Bioconductor) was used to perform the enrichment analysis. The KEGG rest API (https://www.kegg.jp/kegg/rest/keggapi.html (accessed on 5 May 2022)) was used to get the newest gene notations of the KEGG pathway and the R package clusterProfiler (Version 3.14.3, Bioconductor) was used to obtain the enrichment results. The minimum gene was set as 5 and the maximum as 5000; a *p* value < 0.05 and FDR < 0.25 were considered statistically significant.

### 2.4. Construction of the Protein–Protein Interaction Network

The STRING V11.0 database (https://cn.string-db.org/ (accessed on 5 May 2022)) was used to analyze the interactions between the genes and construct a PPI network; after that, the disconnected nodes in the network were hided. The result was downloaded from STRING, then imported into the Cytoscape V3.9.1 software (UC, San Diego, CA, USA) to find the top 10 node genes by the CytoHubba plug-in in 9 ways, and then we took the intersection to filtrate the hub genes. In the CytoHubba plug-in, the MCC is the most accurate method, so we selected the top 2 genes in the MCC score, TP53 (p53) and JUN (c-Jun), as the key genes.

### 2.5. Biochemical Measurement of Blood Samples

All the used blood samples were collected after the pregnant women were admitted to hospital and before delivery. Blood samples were drawn from the antecubital vein in the arm, then centrifuged at 3000 rpm for 20 min, and the serum was collected and stored at −80 °C until processing. The serum concentration of p53 was determined with an ELISA kit from abcam (Human p53 ELISA Kit; ab46067), and the serum concentration of c-Jun was measured by an ELISA kit from Cell Signaling Technology (CST) (FastScan Total c-Jun ELISA Kit; 23176C).

### 2.6. Statistic

The “limma” R package and Cytoscape V3.9.0 software were used to perform the analysis of the public gene expression data. SPSS V22.0 software (SPSS, Chicago, IL, USA) was used to analyze the data of the demographic characteristics of the participants and the biochemical measurements. The normally distributed continuous variables were expressed as the means ± standard deviations and the significance between the means was tested using Student’s *t*-test. Mann–Whitney tests were used to compare non-normally distributed data, with data expressed as the median and interquartile range. The data counting was expressed as a rate (%) by using Chi-square tests. Pearson correlation analysis was used to analyze the relationships between the clinical characteristics and gene expression. Receiver operating characteristic (ROC) curve and area under the curve (AUC) values analyses were performed to evaluate the diagnostic accuracy of the gene expression levels for preeclampsia. A *p* value < 0.05 was considered statistically significant.

## 3. Results

### 3.1. Results

#### 3.1.1. Clinical Features of the Participants

A total of 60 pregnant patients with PE and 60 controls with normal pregnancy were included in this study. There were no significant differences in maternal age, parity, history of PE, chronic hypertension, pre-gestational diabetes mellitus, antiphospholipid syndrome, preexisting kidney disease, and familiar risk (*p* > 0.05). Additionally, the PE group had a higher body mass index (BMI), maximum systolic blood pressure (SBP), maximum diastolic blood pressure (DBP), mean artery pressure (MAP), and higher urinary protein levels; babies born from the PE group had a lower fetal birth weight than those from the control group (*p* < 0.05) (Table 1).

#### 3.1.2. Identification of DEGs in Blood Samples

**Figure 1:**
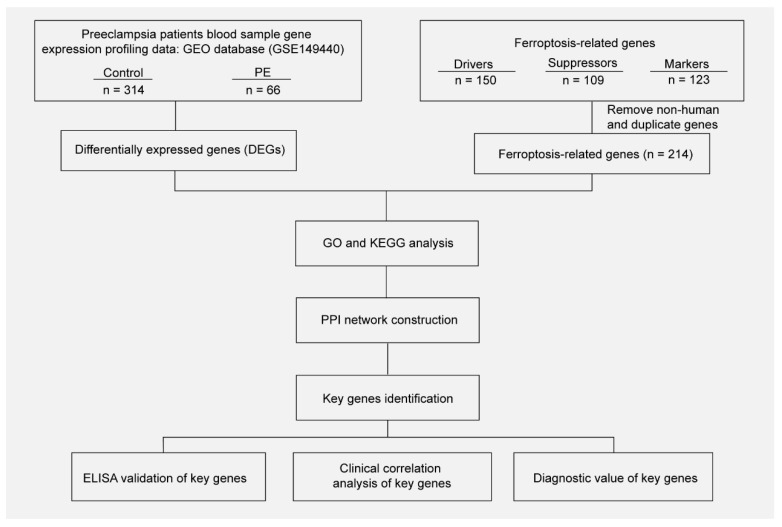
Workflow of searching hub genes in PE.

**Figure 2:**
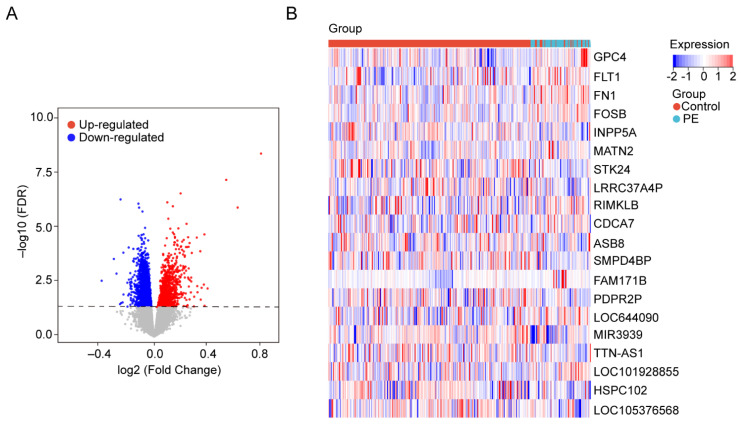
Identification of DEGs in the blood samples. (**A**) The volcanic maps. Red dots represent upregulated genes; blue dots represent downregulated genes, and gray dots represent no significance genes. (**B**) Heatmap for hierarchical clustering of DEGs in the control group and PE group.

The gene expression data in blood samples from normal pregnancy and patients with PE were downloaded from dataset GSE149440 in the Gene Expression Omnibus (GEO). The dataset contains a total of 735 expression data, including 189 preterm premature rupture of membranes (PPROM), 166 spontaneous preterm delivery (sPTD), 314 control, and 66 early-preeclampsia blood samples (Figure 1). The DEGseq R package was used to perform the data normalization and differential gene expression analysis. The results showed that there was a total of 5913 DEGs, including 1234 upregulated genes and 4679 downregulated genes. The DEGs were visualized using a volcano plot (Figure 2A). The top 30 upregulated genes are shown in Table 2 and the top 30 downregulated genes are shown in Table 3. MIR3939, LOC105376568, TTN-AS1, STK24, INPP5A, HSPC102, SMPD4BP, LRRC37A4P, PDPR2P, and ASB8 were the top 10 upregulated genes. FAM171B, FOSB, FLT1, FN1, RIMKLB, MATN2, GPC4, LOC101928855, LOC644090, and CDCA7 were the top 10 downregulated genes. The expression of the top 20 DEGs listed by *p* values is shown by the heatmap (Figure 2B).

#### 3.1.3. GO and KEGG Pathway Enrichment Analysis of the DEGs

**Figure 3:**
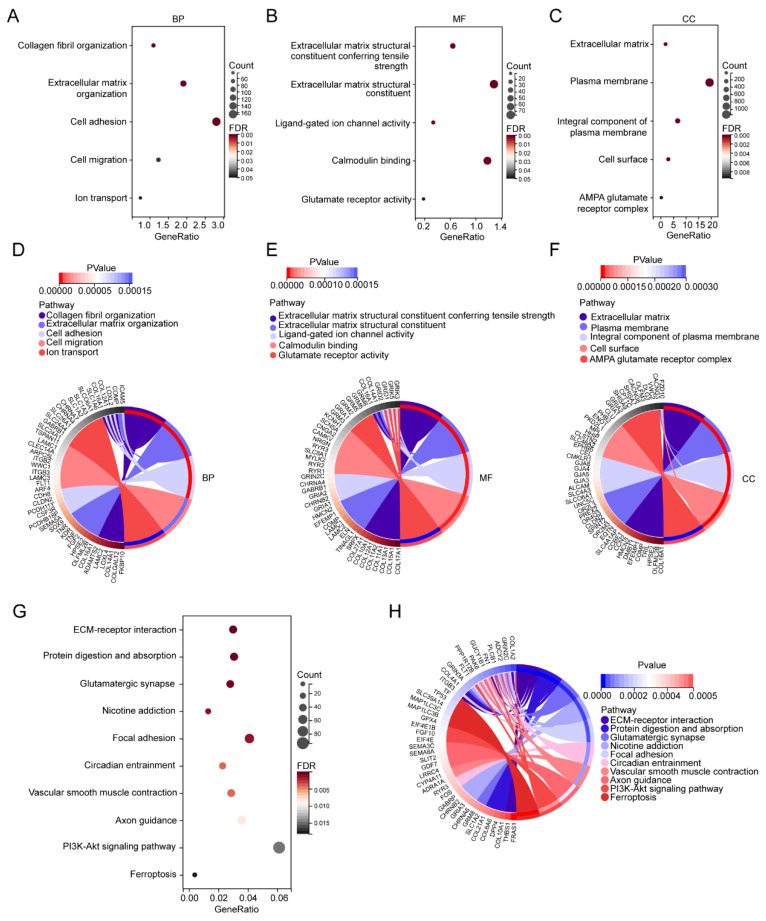
GO and KEGG enrichment analyses of the DEGs. (**A**) Bubble plots of the biological process of the GO analysis. (**B**) Bubble plots of the molecular function of the GO analysis. (**C**) Bubble plots of the cellular component of the GO analysis. (**D**) Circle plot and network visualizing the biological process of the GO analysis. (**E**) Circle plot and network visualizing the molecular function of the GO analysis. (**F**) Circle plot and network visualizing the cellular component of the GO analysis. (**G**) Bubble plot of the KEGG pathway enrichment of the DEGs. (**H**) Circle plot and network visualizing the KEGG pathway.

To investigate the biological functions of these DEGs, GO enrichment analysis was performed. The GO enrichment analysis results for the biological process (BP) showed that DEGs were mainly enriched in collagen fibril organization, extracellular matrix organization, cell adhesion, cell migration, and ion transport (Figure 3A,D). Extracellular matrix structural constituent conferring tensile strength, extracellular matrix structural constituent, ligand-gated ion channel activity, calmodulin binding, and glutamate receptor activity were enriched in Molecular Function (MF) (Figure 3B,E). For cellular component (CC), the DEGs were mainly enriched in the extracellular matrix, plasma membrane, integral component of plasma membrane, cell surface, and AMPA glutamate receptor complex (Figure 3C,F). The KEGG pathway found that the DEGs were mainly enriched in the ECM–receptor interaction, protein digestion and absorption, glutamatergic synapse, nicotine addiction, focal adhesion, circadian entrainment, vascular smooth muscle contraction, axon guidance, PI3K-Akt signaling pathway, and ferroptosis (Figure 3G,H).

**Figure 4:**
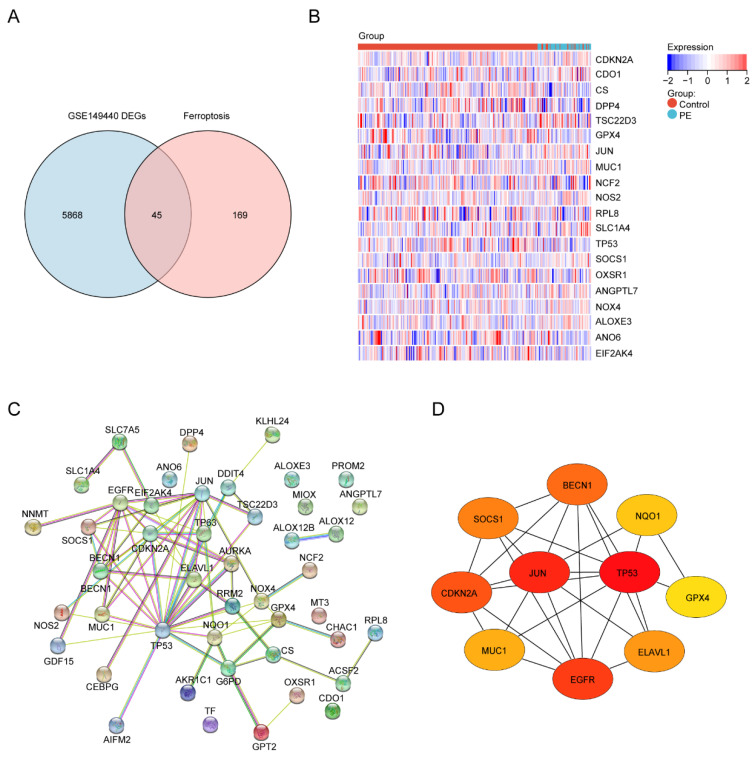
Identification of the ferroptosis-related DEGs and the identification of candidate ferroptosis-related hub genes in the blood samples of PE. (**A**) Venn diagram of the ferroptosis-related DEGs. (**B**) Heatmap depicting the expression levels of the top 20 ferroptosis-related DEGs. (**C**) PPI network analysis of the ferroptosis-related DEGs. (**D**) Ten hub genes were selected by the CytoHubba plug-in in Cytoscape.

Next, to identify the ferroptosis DEGs, 214 ferroptosis-related genes were downloaded from FerrDb and intersected with the DEGs from GSE149440. The Venn plot shows the intersection of DEGs and ferroptosis-related genes (Figure 4A). In total, 45 ferroptosis-related DEGs were found, including 15 upregulated and 30 downregulated genes (Table 4). The expression levels of the top 20 ferroptosis-related DEGs listed by *p* values in two groups are displayed by the heatmap hierarchical clustering (Figure 4B). Finally, the STRING online database was used to analyze the ferroptosis-related DEGs, and a PPI network with 45 nodes and 73 edges was obtained (Figure 4C). Finally, ten hub genes were selected using the CytoHubba plug-in in Cytoscape (Figure 4D, Supplement Table S1).

#### 3.1.4. Hub Gene Expression and Correlation with MAP

**Figure 5:**
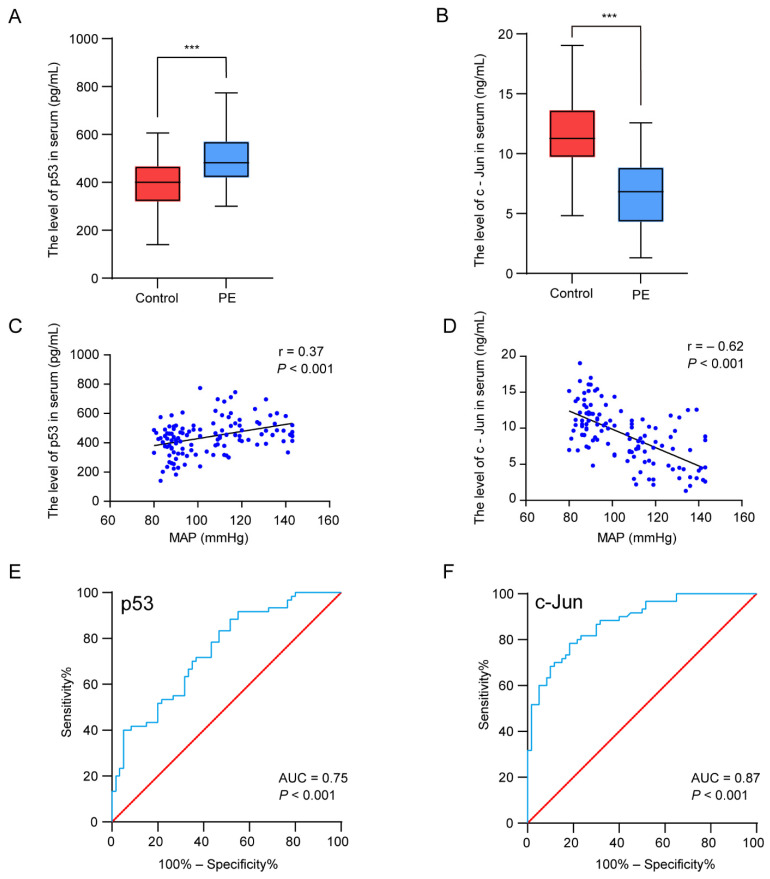
Experimental verification of the two hub genes, correlation analysis between the hub genes’ expression levels and clinical features, and the diagnostic accuracy analysis. (**A**,**B**) The concentration of p53 (**A**) and c-Jun (**B**) in serum. (**C**,**D**) Pearson correlation plots between MAP and p53 (**C**) and c-Jun (**D**). (**E**,**F**) The ROC curves were plotted to examine the diagnostic potential of p53 (**E**) and c-Jun (**F**). *** *p* < 0.001.

We next verified the serum p53 and c-Jun levels in patients with PE and the controls by ELISA. The results suggested that the level of p53 was significantly higher (*p* < 0.001) in the PE patients, whereas the serum c-Jun concentrations decreased (*p* < 0.001) (Figure 5A,B). To further investigate the relationship between the hub genes and the clinical features of the patients, Pearson correlation was used and revealed a statistically significant correlation between the mean arterial blood pressure (MAP) and p53 (r = 0.37, *p* < 0.001) and c-Jun (r = −0.62, *p* < 0.01) (Figure 5C,D). Regarding the potential of the markers for discriminating PE patients from normal pregnant women, the ROC analyses revealed that the AUC values of p53 and c-Jun were 0.75 and 0.87, respectively (Figure 5E,F). The results indicate that p53 and c-Jun have a medium diagnostic value and have the potential to be used as diagnostic markers in PE.

## 4. Discussion

Ferroptosis, a newly described type of programmed cell death driven by iron-dependent phospholipid peroxidation, has been shown to be involved in a range of diseases [32]. Several lines of evidence have indicated the widespread existence of ferroptosis in some diseases, including degenerative diseases, carcinogenesis, stroke, and traumatic brain injury [33]. Recently, there has been increasing evidence that ferroptosis plays a crucial role in the initiation and development of PE [21,24,34,35]. Therefore, ferroptosis related biomarkers may provide potential diagnostic biomarkers and therapeutic targets for pregnant women with PE. In the present study, the whole blood transcriptome in PE patients were extracted from the GEO database. Gene expression analysis identified 5913 DEGs in PE samples as compared to normal samples. Next, all DEGs were subjected to GO enrichment analysis and KEGG pathway enrichment analysis. The KEGG pathway enrichment analysis revealed that the ferroptosis were enriched. Biovenn was utilized to get the intersection of the DEGs and the ferroptosis-related genes, and finally 45 ferroptosis-related DEGs were identified. Finally, PPI analysis was performed and the key genes p53 and c-Jun were identified and validated. In conclusion, our study found that p53 and c-Jun have diagnostic value for PE.

One of the guardians of the genome, p53, is the most widely studied tumor suppressor gene, and p53 mutations are frequently studied with regard to cancer risk [36]. Therefore, maintaining the function and stability of p53 is important for cellular homeostasis. Since a study linking p53 to ferroptosis regulation was first reported in 2015, more than 170 studies on p53 and ferroptosis have been published [37]. Ferroptosis is characterized by increased lipid peroxidation and ROS due to metabolic dysfunction. The primary function of p53 is to mediate cellular and systemic metabolism. Numerous studies have found that p53 is closely related to all key metabolic pathways involved in ferroptosis [38]. In addition, p53-mediated ferroptosis appears to be a barrier to cancer development, as it can inhibit tumor formation independently of p53-mediated cell apoptosis, senescence, and cycle arrest. Studies have shown that, in addition to downregulating SLC7A11 and impairing GSH biosynthesis, p53 promotes ferroptosis by regulating other metabolic pathways [39]. Recent studies have indicated that p53 is involved in the development of preeclampsia [40,41,42,43]. In our profiling study, we confirmed the increased expression of p53 in PE patient serum. These results suggest that p53 may be involved in the regulation of ferroptosis in PE, but the specific molecular mechanism needs further experimental confirmation.

The c-Jun transcription factor was the first oncogenic transcription factor discovered, and is the cellular homologue of v-Jun [44]. Studies have found that c-Jun with physiological functions can promote embryonic liver development and liver and skin regeneration [45]. Moreover, c-Jun is implicated in carcinogenesis and development of many tumors. During tumorigenesis, c-Jun is known as an important regulator of major biological events, such as cell proliferation, by specifically regulating EGFR, KGF, CyclinD1, and other proliferation-stimulating genes [46]. Some studies found that c-Jun also downregulates p53, thereby inhibiting apoptosis. Recently, a growing number of research has indicated that c-Jun participates in the regulation of ferroptosis processes in some tumors [47,48,49]. Studies have shown that c-Jun can inhibit ferroptosis by stimulating GSH synthesis by increasing PSAT1 and CBS transcription [47]. In this study, we found decreased expression of c-Jun in the whole blood of the PE patients. Our results suggest that the anti-ferroptosis effect of c-Jun may be suppressed to some extent in patients with PE. However, the specific molecular mechanism by which c-Jun regulates ferroptosis needs to be further explored in PE.

There are some limitations to our study, as well. First, the potential ferroptosis-related biomarkers identified in this study still need further literature support and laboratory evidence validation. Second, ferroptosis-related genes are derived from the constantly updated FerrDb, and there are more genes to be discovered. Finally, the sample size was relatively small and larger samples are required for the further validation of p53 and c-Jun as a biomarker for PE.

## 5. Conclusions

In the present study, we explored the crucial genes of blood ferroptosis-related biomarkers by bioinformatics methods. We identified two key genes, p53 and c-Jun, associated with ferroptosis in PE, which may distinguish PE patients from normal pregnant women, and may be potential ferroptosis-related biomarkers for disease diagnosis and treatment monitoring.

## 6. Patents

There are no patents involved in this study.

## Figures and Tables

**Table 1:** Demographic characteristics of the participants in this study.

Variable	Control Group (*n* = 60)	PE Group (*n* = 60)	*p* Value
Maternal age, y	29 (26–33)	29 (27–33)	0.819
Body mass index, kg/m^2^	21 (19–22)	22 (20–25)	0.006
Parity, *n* (%)			0.355
Primipara	32 (53)	38 (63)	
Multipara	28 (47)	22 (37)	
History of the PE, *n* (%)	3 (5)	4 (7)	1.000
Chronic hypertension, *n* (%)	0 (0)	2 (3)	0.496
PGDM, *n* (%)	2 (3)	2 (3)	1.000
Antiphospholipid syndrome, *n* (%)	1 (2)	2 (3)	1.000
Preexisting kidney disease, *n* (%)	0 (0)	2 (3)	0.496
Familiar risk, *n* (%)	1 (2)	3 (5)	0.619
Systolic pressure, mmHg	117 (112–126)	161 (146−170)	<0.001
Diastolic pressure, mmHg	75 (71–79)	98 (92–114)	<0.001
MAP, mmHg	89 (86–93)	119 (111–132)	<0.001
24-h proteinuria, g/24 h	0.05 (0.04–0.12)	1.33 (0.64–3.43)	<0.001
Gestational age at birth, d	266 (255–276)	259 (244–267)	<0.001
Fetal birth weight, g	3194 (2949–3275)	2900 (2200–3200)	0.002
RDS, *n* (%)	1 (2)	16 (27)	<0.001
NICU, *n* (%)	1 (2)	16 (27)	<0.001

Data are presented as the median (interquartile range) and mean ± standard deviation unless indicated as *n* (%). PE = preeclampsia; PGDM = pre-gestational diabetes mellitus; MAP = mean artery pressure; RDS = respiratory distress syndrome; NICU = neonatal intensive care unit. A *p* value < 0.05 was considered significant.

**Table 2:** Top 30 upregulated genes of the DEGs.

Gene Symbol	Log_2_FC	*p* Value
MIR3939	0.681836871	0.0000000065
LOC105376568	0.459679751	0.0000000968
TTN-AS1	0.168772851	0.0000003906
STK24	0.084107410	0.0000009831
INPP5A	0.119524431	0.0000014759
HSPC102	0.532569188	0.0000016817
SMPD4BP	0.090404584	0.0000053122
LRRC37A4P	0.206650798	0.0000090054
PDPR2P	0.154363661	0.0000144329
ASB8	0.103036862	0.0000148492
GLYR1	0.119425589	0.0000221570
SLC25A6P2	0.127278093	0.0000262861
ERV3-1	0.320543342	0.0000268127
TOR1A	0.132429450	0.0000346933
WHAMMP1	0.216623800	0.0000359022
LRRC37A17P	0.271869224	0.0000371765
DHDDS-AS1	0.132565620	0.0000379569
MAX	0.080250037	0.0000396618
SELP	0.188509826	0.0000471182
RPS2P7	0.148369736	0.0000508025
NQO2	0.178847959	0.0000565570
FAM153A	0.129231134	0.0000603660
TNFSF14	0.132533146	0.0000616615
PDPR	0.225867791	0.0000652686
SLC4A10	0.146662353	0.0000768642
TAB3	0.127250551	0.0000785952
COLQ	0.070045308	0.0000844558
PDLIM1	0.149533133	0.0000849043
OR6K3	0.172019882	0.0000919229
NFE4	0.219922283	0.0000964147

**Table 3:** Top 30 downregulated genes of the DEGs.

Gene Symbol	Log_2_FC	*p* Value
FAM171B	−0.214300119	0.0000007277
FOSB	−0.100125939	0.0000011258
FLT1	−0.095896809	0.0000016907
FN1	−0.074430719	0.0000025301
RIMKLB	−0.057612207	0.0000136380
MATN2	−0.067397889	0.0000264791
GPC4	−0.079923918	0.0000285482
LOC101928855	−0.099017279	0.0000309617
LOC644090	−0.085740243	0.0000373470
CDCA7	−0.056959849	0.0000398187
LOC101059954	−0.078430951	0.0000413221
LINC02487	−0.078594043	0.0000481066
ELOC	−0.037768067	0.0000496455
NHLH2	−0.066026726	0.0000582698
LOC100506489	−0.064018314	0.0000697048
LOC107986383	−0.077890143	0.0000722787
TUBB6	−0.080615791	0.0000987167
PRG1	−0.162206377	0.0001033506
LINC00309	−0.054808819	0.0001114693
TCN2	−0.099952456	0.0001141195
LOC112267901	−0.132601517	0.0001190145
SERINC2	−0.106660347	0.0001205236
IER5L-AS1	−0.084976169	0.0001325151
LOC284912	−0.091842494	0.0001444731
CHRNB3	−0.058923918	0.0001519665
NR4A2	−0.063301192	0.0001692061
YEATS4	−0.196128294	0.0001758740
LINC02953	−0.069851232	0.0001776020
LOC101928517	−0.077337704	0.0001810568
SNORD114-7	−0.075879892	0.0001837738

**Table 4:** Intersection of the DEGs and ferroptosis-related genes.

Gene Symbol	Log_2_FC	*p* Value
CS	0.088024037	0.0013860456
GPX4	0.127606332	0.0033606329
DPP4	0.115610006	0.0041819650
ANO6	0.092445408	0.0056013748
NCF2	0.102692749	0.0106923591
OXSR1	0.073276793	0.0132039673
RPL8	0.070519563	0.0163624365
EIF2AK4	0.051333877	0.0219376884
TP53	0.061276552	0.0227802882
ALOX12	0.090642503	0.0238840276
G6PD	0.06437918	0.0245358858
ELAVL1	0.047106006	0.0257012638
BECN1	0.054079622	0.0360843333
SLC7A5	0.141674054	0.0444067288
KLHL24	0.05860919	0.0484929326
SLC1A4	−0.046713571	0.0025230647
CDO1	−0.042790266	0.0021886411
ALOXE3	−0.052995436	0.0044745713
ANGPTL7	−0.053769764	0.0069292813
JUN	−0.049405558	0.0081302033
SOCS1	−0.087858572	0.0098413330
NOX4	−0.027259999	0.0153337346
NOS2	−0.039584599	0.0194259690
CDKN2A	−0.033970203	0.0216855291
TSC22D3	−0.044797939	0.0219355680
MUC1	−0.045560811	0.0225179167
AURKA	−0.051276727	0.0233380462
CHAC1	−0.050305866	0.0235809423
NQO1	−0.036099395	0.0257095124
TP63	−0.026632072	0.0308774916
GPT2	−0.043220744	0.0311273224
AIFM2	−0.036737201	0.0329521820
MT3	−0.04429197	0.0335564152
ACSF2	−0.028855554	0.0337071772
EGFR	−0.032015545	0.0353011283
GDF15	−0.0688667	0.0360222189
ALOX12B	−0.04162569	0.0379310322
MIOX	−0.039351283	0.0409310117
NNMT	−0.044199194	0.0411587687
TF	−0.027938216	0.0417271472
CEBPG	−0.039507462	0.0428706382
RRM2	−0.061984651	0.0429017623
AKR1C1	−0.044489901	0.0430243813
PROM2	−0.037538817	0.0459449958
DDIT4	−0.044721922	0.0483266707

## Data Availability

Publicly available data sets were analyzed in this study. Publicly available data sets were analyzed in this study.

## Supplementary Materials

The following supporting information can be downloaded at: https://www.mdpi.com/article/10.3390/biology11070950/s1, Supplement Table S1. Top 10 node genes by 9 topological analysis methods of CytoHubba.
