# Supplementary material for: Ferroptosis-Related Proteins Are Potential Diagnostic Molecular Markers for Patients with Preeclampsia
Source: Biology (Basel). 2022 Jun 22;11(7):950. doi: 10.3390/biology11070950 (PMC9311911; doi:10.3390/biology11070950)
Supplement: Supplementary file 1 [file biology-11-00950-s001.zip › biology-1757473-supplementary.pdf]

Supplement Table S1. Top 10 node genes by 9 topological analysis methods of CytoHubba.

MCC	MNC	Degree	EPC	BottleNeck	Closeness	Radiality	Betweenness	Stress
TP53	TP53	TP53	TP53	TP53	TP53	TP53	TP53	TP53
JUN	JUN	JUN	JUN	JUN	JUN	JUN	G6PD	EIF2AK4
EGFR	EGFR	EGFR	EGFR	G6PD	EGFR	NQO1	JUN	G6PD
CDKN2A	CDKN2A	CDKN2A	CDKN2A	EIF2AK4	NQO1	EGFR	CS	JUN
BECN1	BECN1	BECN1	BECN1	CS	CDKN2A	ELAVL1	EIF2AK4	CS
SOCS1	ELAVL1	NQO1	ELAVL1	EGFR	BECN1	G6PD	NQO1	SLC7A5
ELAVL1	NQO1	ELAVL1	NQO1	CDKN2A	ELAVL1	BECN1	EGFR	ACSF2
MUC1	SOCS1	GPX4	SOCS1	GPT2	G6PD	CDKN2A	RRM2	RRM2
NQO1	TP63	G6PD	TP63	GPX4	GPX4	GPX4	GPX4	ELAVL1
GPX4	AURKA	NOX4	MUC1	NOX4	NOX4	RRM2	NOX4	NQO1
